# NO_2_ Sensing Properties of Cr_2_WO_6_ Gas Sensor in Air and N_2_ Atmospheres

**DOI:** 10.3389/fchem.2019.00907

**Published:** 2020-01-23

**Authors:** Yi Wu, Meng Yan, Chen Tian, Yuhang Liu, Zhongqiu Hua

**Affiliations:** ^1^Tianjin Key Laboratory of Electronic Materials and Devices, School of Electronics and Information Engineering, Hebei University of Technology, Tianjin, China; ^2^Institute of Polymer Materials, Beijing Institute of Machine and Equipment, Beijing, China

**Keywords:** nitrogen dioxide, Cr_2_WO_6_, low dimensional material, gas sensors, humidity

## Abstract

Gas sensors were fabricated from Cr_2_WO_6_ nanoparticles for NO_2_ detection. Low dimensional materials Cr_2_WO_6_ were prepared by a wet chemistry method followed by hydrothermal treatment. The morphology of the nanoparticles and their sensing properties to NO_2_ were investigated in both dry and humid conditions. Additionally, the sensing response was also characterized in a non-oxygen condition. It was concluded that the sensor responses in N_2_ conditions were higher than that in air conditions at 200°C. Moreover, the sensing characteristics were inhibited by water vapor at 200°C. The oxygen adsorption behavior was also investigated to verify the basic sensing mechanism of Cr_2_WO_6_ in the absence and presence of NO_2_ and water vapor separately. Based on the power law response, it was indicated that both NO_2_ and water vapor have a strong adsorption ability than oxygen ions of Cr_2_WO_6_ sensors.

## Introduction

National nitrogen oxide (NO_x_) emissions were 20.6 million metric tons (Mt) in 2015, with annual growth rates of 5.9% since 1949 in China (Richter et al., 2005; Sun et al., 2018). Among them, owing to its toxic effects to animals and plants, NO_2_ is irritant and corrosive even at ppm level with serious harm to the respiratory tract and causticity particularly in children and elderly (Ling and Leach, 2004; Kida et al., 2009). Thus, determination of NO_x_ emissions is vital to regional and global ozone air pollution, acid deposition, and climate change (Jaegle et al., 2005). There have been great demands for cheap, reliable, and effective methods of real-time monitoring of NO_2_ level in the environment (Stǎnoiu et al., 2012). To date, gas sensors based on metal oxide semiconductors (MOS) have been extensively investigated and successfully commercialized for NO_x_ detection due to their superior properties, simple structure, and low cost (Afzal et al., 2012). Among them, WO_3_ based sensors show an excellent property to NO_2_ in respect of sensitivity (Choi et al., 2004; Hua et al., 2018d). It was reported that WO_3_ sensors fabricated through a wet process were sensitive to ppb levels of NO_2_ with a low temperature due to a strong adsorption ability of NO_2_ onto tungsten atoms compared with the weak adsorption of oxygen (Choi et al., 2004). Recently, gas sensors based on P-type MOS materials have been reported to have a sensitive and selective response to NO_2_ with a low cross-sensitivity to humidity (Nguyen and El-Safty, 2011; Stǎnoiu et al., 2012). It was reported that the NO_2_ sensors based on Cr_2_O_3_ showed a selectivity relative to CO and low cross-sensitivity to humidity, which was due to the higher surface reactivity toward NO_2_ than CO through the electrical resistance and work function changes both in dry and humid air (Stǎnoiu et al., 2012). Gas sensors based on NiO nanosheets were fabricated by a hydrothermal method, which showed high sensitivity and selectivity to NO_2_ (Nguyen and El-Safty, 2011). That may be due to NO_2_ having higher electron affinity than the pre-adsorbed oxygen (Hoa et al., 2009). In this paper, chromium tungstate (Cr_2_WO_6_) was prepared by a wet chemistry method followed by hydrothermal treatment. The sensing properties to NO_2_ in oxygen and non-oxygen atmosphere were investigated under dry and humid conditions, respectively. It was found that sensors based on P-type Cr_2_WO_6_ were very sensitive to NO_2_ even with a high humid condition. In addition, the sensing mechanism was verified by the oxygen adsorption behavior in different conditions. Eventually, the fundamental sensing mechanism to NO_2_ was explained.

## Experimental

**Figure 1:**
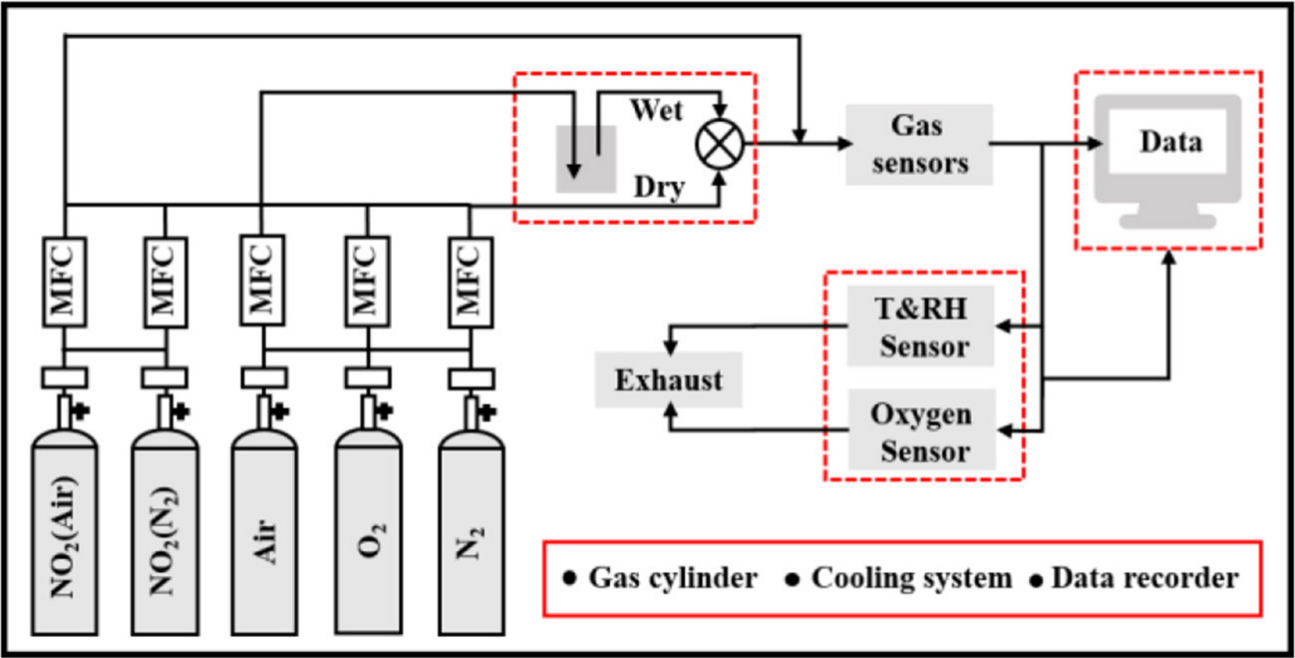
Experimental system for the sensing characteristics of sensor devices.

Chromium(III) nitrate nonahydrate (99.95%) and sodium tungstate dehydrate (ACS 99.0–101.0%) were provided by Shanghai Aladdin Biochemical Technology Co., Ltd. Chromium tungstate (Cr_2_WO_6_) nanoparticles were synthesized by a hydrothermal assisted process as described in Supplementary Materials A (Zhou et al., 2015). Subsequently, the powders were annealed at 1,000°C in air for 2 h. The samples were characterized using X-ray diffraction (XRD; D8 FOCUS, Bruker, Germany) in Cukα radiation with corresponding wavelengths of 1.54 Å and a filament current and voltage of 15 mA and 40 kV and a field-emission scanning electron microscope (FE-SEM; Nova Nano SEM 450, FEI). The surface morphology of the material was analyzed by transmission electron microscopy (TEM; Tecnai-F20, FEI, USA) with an accelerating voltage of 200 kV. The sample powders were mixed with glycerin to form a homogeneous paste and then was screen-printed on the alumina substrate. In order to obtain good stability, all sensors were aged at 400°C for 24 h. The gas sensing performance was measured by DC resistance with a homemade apparatus equipped with a dynamic gas distribution system as shown schematically in Figure 1. Target gases were supplied by gas cylinders with appropriate concentrations balanced with the carrier gases (air or nitrogen). The humidity and oxygen concentrations were calibrated using a humidity sensor (SHT31-ARP, Sensirion, Switzerland) and oxygen analyzer (SST, England), respectively. Keithley multimeter (Keithley 2000, USA) was used to record all data in real time. The sensor response was defined as *S* = *R*_a_*/R*_g_, where *R*_a_ and *R*_g_ were the resistances in the presence of air/N_2_ and oxidizing gases, respectively.

## Results and Discussion

**Figure 2:**
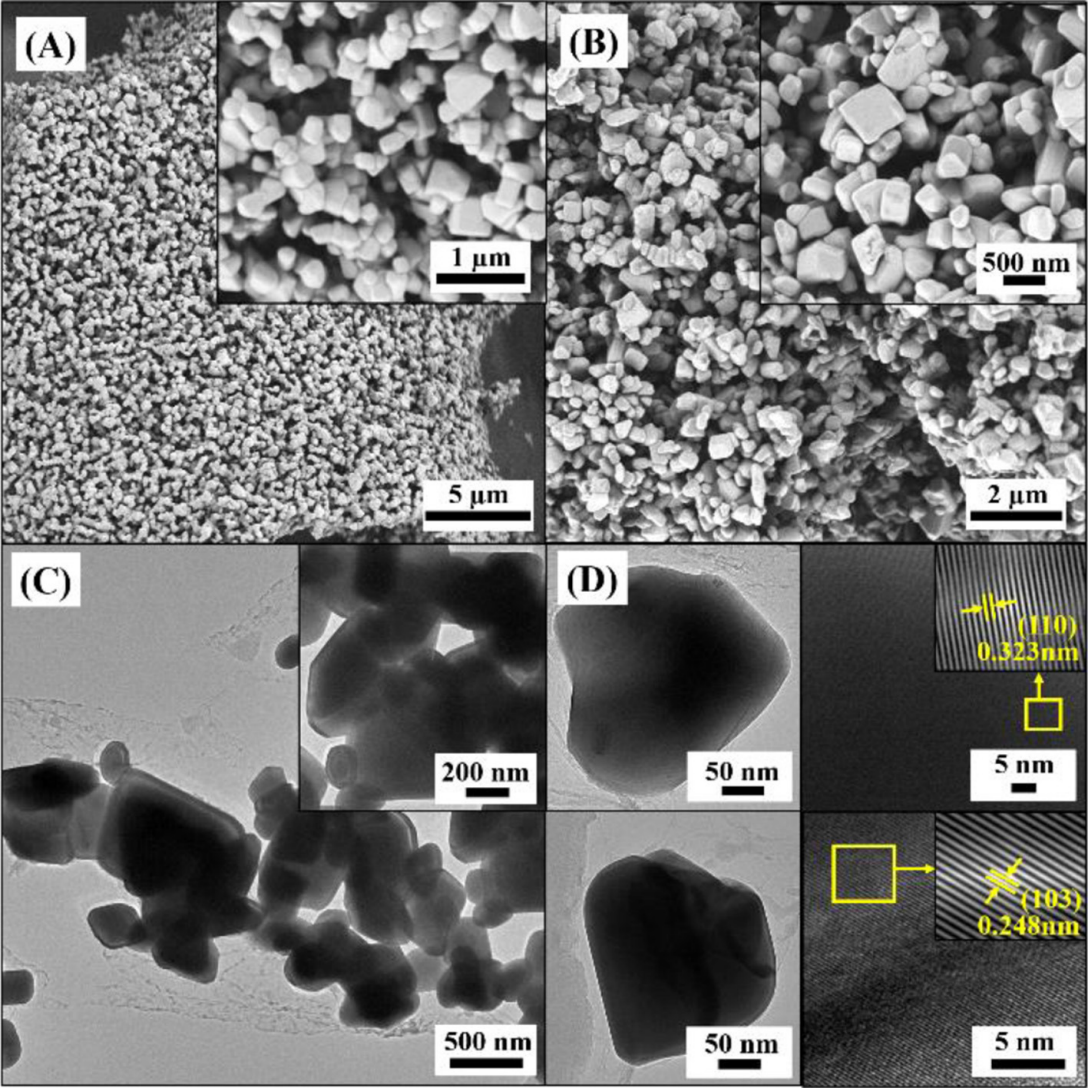
The morphology analysis of powders **(A,B)** SEM images, **(C)** TEM images, and **(D)** HRTEM micrograph with lattice diffraction pattern of nanoparticles.

In order to obtain a high purity of Cr_2_WO_6_ phase, a high sintering temperature of 1,000°C was used and the crystal structure was characterized by XRD. Figure S3 shows the XRD patterns of prepared Cr_2_WO_6_ powders. Obviously, XRD patterns exhibit very narrow peaks indicating a good crystal quality and the patterns could be well-fit with the tetragonal phase (JCPDS 35-0791) indicating a high purity of the prepared Cr_2_WO_6_. The morphology of Cr_2_WO_6_ powders was characterized by SEM and TEM. Figures 2A,B show SEM images of Cr_2_WO_6_ powders, which consisted of huge number of particles in a grain shape with a large size. According to the insert SEM images the grain shape is estimated around 300 nm by counting. TEM images in Figure 2C show that the grain size is consistent with the SEM images. Additionally, HRTEM images in Figure 2D also suggest a good crystalline quality of Cr_2_WO_6_ nanoparticles and the lattice spacing of 0.323 nm and 0.248 nm is in good accordance with (110) and (103) planes of tetragonal Cr_2_WO_6_ (JCPDS 35-0791) and results of XRD (Zhou et al., 2015).

**Figure 3:**
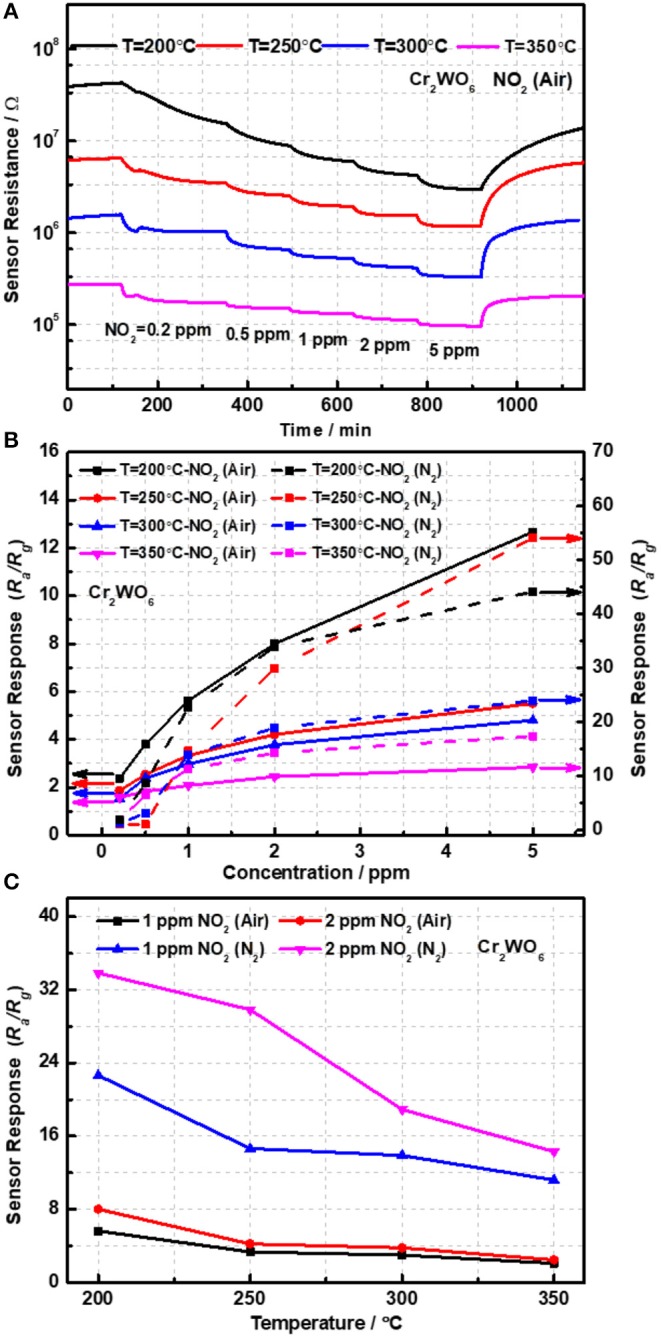
**(A)** The transient response of sensor devices and **(B)** the sensor responses as a function of NO_2_ concentration and **(C)** temperatures.

The sensing properties were characterized with NO_2_ ranging from 0.2 to 5 ppm balanced with syntheses with an operation temperature of 200–350°C. According to previous reports, chemiresistive type sensors with a low concentration of carriers, i.e., holes in the present case, have a strong transduction ability with high cost of resistance (Bârsan et al., 2010; Hua et al., 2018a). It is worth noting that two identical sensor devices fabricated from the same materials were measured; however, only one sensors' data was presented for simplicity. The other one was used as a reference and not shown. Figure 3A presented the time and temperature dependence of sensor resistance. It was found that sensor resistance significantly increased with reduction in operation temperatures. When the temperature decreased from 250 to 200°C, sensor resistance was almost increased by 3 times. However, when the temperature was lower than 200°C, sensors gave an extremely high resistance reaching to 10^8^ Ω and over the range of measurement (Keithley 2000), which is also very difficult for practical applications. Thus, sensing response was only characterized from 200 to 350°C. One can note that sensor resistance is significantly reduced when exposed to NO_2_, suggesting a P-type response of Cr_2_WO_6_. Moreover, the sensor responses were found to be highly temperature-dependent and increased with reduction in temperatures (Bodneva et al., 2019). When the working temperature increased to 350°C, the responses of 5 ppm NO_2_ were reduced by 4.5 times.

Detection of NO_2_ in the non-oxygen atmosphere was highly required and could be applied to some specific scenes, such as the fuel leaks in aerospace systems. The sensor responses to NO_2_ balanced with N_2_ were investigated from 200 to 350°C in the absence of oxygen. Owing to its p-type conduction, the baseline resistance in N_2_ was largely enhanced caused by the increase in electrons released by adsorbed oxygens on the surface. Consequently, the sensor responses were greatly increased to NO_2_ higher than 1 ppm and much higher than in air atmosphere as shown in Figure 3B. It was shown that the responses in N_2_ conditions were 4 times as much as that in air conditions at 200°C as shown in Figure 3C. It was well-known that in the presence of oxygen there could be a competitive adsorption between oxygen and NO_2_, and therefore, that may give a reason for the clear difference of sensing responses in the presence and absence of oxygen (Hua et al., 2018d). What's more, the competitive relation between NO_2_ and oxygen will be analyzed by the oxygen adsorption behavior in the following. In addition, it was indicated that the sensing responses in N_2_ atmosphere were highly dependent on the working temperatures similar to that in the air atmosphere. When the temperature decreased from 350 to 200°C, sensor resistance was almost promoted by 2 orders and sensor responses were enhanced by almost 4 times. As shown in Figure S4, it seems that the sensors gave a relatively poor response to NO_2_ below 1 ppm, almost no response in a dry condition. In other words, the limit of detection (LOD) of NO_2_ for Cr_2_WO_6_ nanoparticles is better than 1 ppm. This is very similar to a typical n-type MOS gas sensor when exposed to reducing gases such H_2_ and CO (Hua et al., 2018c). A small concentration of gas could be shielded by adsorption of oxygen due to the release of free electrons by the reaction of adsorbed oxygens with reducing gases. Thus, small concentration of reducing gases could not be detected (Hua et al., 2018d). However, in the present case, it is believed that the shielding effect of NO_2_ could be caused by a competitive adsorption of oxygen and NO_2_ on the surface of Cr_2_WO_6_. When NO_2_ adsorbed onto the surface and shared the same sites with oxygen adsorption leading to desorption of oxygen, there was no electron charge transfer. As NO_2_ molecular trap the electrons, which released from desorption of O_2_, the surface density will not generate a net increase. As a result, there was no resistive response, which was known as a chemical shielding effect for MOS gas sensors (Hua et al., 2018d). Finally, a comparison of MOS sensors to NO_2_, including various n-type and p-type materials, was given in Table 1 (Ling and Leach, 2004; Hoa et al., 2009; Nguyen and El-Safty, 2011; Vyas et al., 2013; Marichy et al., 2015). In conclusion, the Cr_2_WO_6_ sensors have reat sensitivity and lower operation temperatures to NO_2_ compared with the n-type materials including SnO_2_/WO_3_ and ZnO both in air and N_2_ atmospheres. Furthermore, some typical p-type materials were listed in Table 1, and the Cr_2_WO_6_ sensors displayed high sensitivity, which had great potential to be applied to some specific scenes. The stability of sensors have been also investigated in Figure S7 of Supplementary Material E.

**Table 1:** The response to NO_x_ compared with other MOS sensors.

**MOS sensors**	**Target gases**	**Detection concentration (ppm)**	**Response**	**Temperature (**°**C)**	**Conduction characteristic**	**References**
SnO_2_/WO_3_	NO_2_/Air	2	6.5	300	n-type	Ling and Leach, 2004
Cr_2_O_3_	NO_2_/Air	3	2	200	p-type	Stǎnoiu et al., 2012
NiO	NO_2_/Air	1	13	250	p-type	Nguyen and El-Safty, 2011
SnO_2_ – SWNT	NO_x_/Air	60	2,300%	200	n-type	Hoa et al., 2009
TiO_2_/CNT700	NO_2_/Air	8	10	150	p-type	Marichy et al., 2015
ZnO	NO_2_/N_2_	20	1.2	300	n-type	Vyas et al., 2013
Cr_2_WO_6_	NO_2_/Air	2	8	200	p-type	This work
Cr_2_WO_6_	NO_2_/N_2_	2	33.9	200	p-type	This work

**Figure 4:**
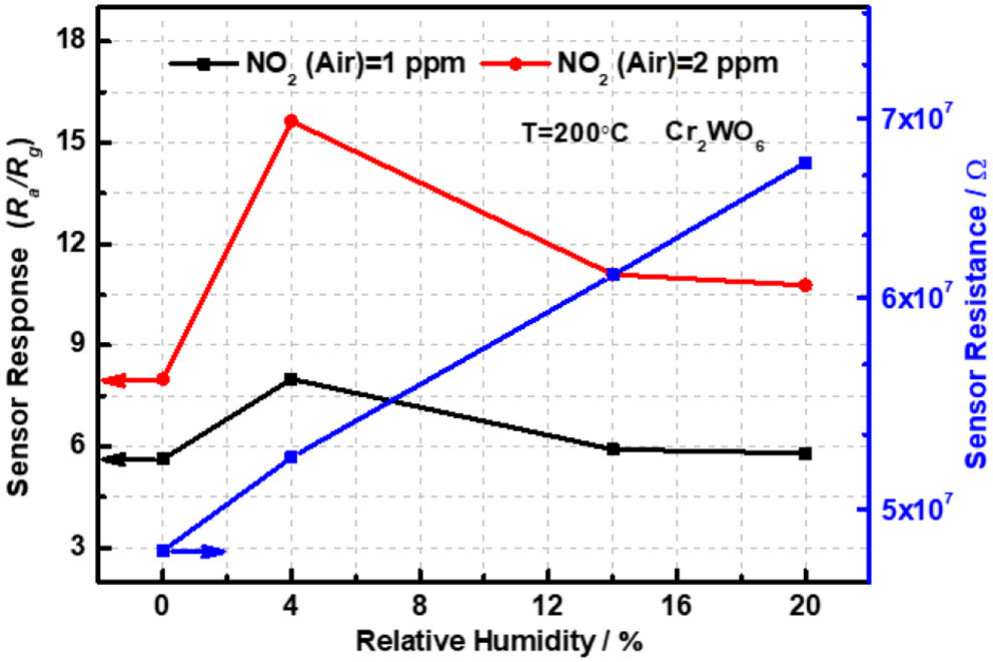
The sensor responses of Cr_2_WO_6_ as a function of humidity and NO_2_ concentration balanced with air or N_2_ at 200°C.

Moreover, the sensing properties to NO_2_ balanced with air in the presence of humidity were also investigated at 200°C and the transient responses were shown in Figure S5 of Supplementary Materials C. It was clear that all sensor resistances were a direct proportion to the relative humidity at 25°C (RH at 25°C) compared with that in dry conditions as revealed in Figure 4. The phenomenon can be explained by water vapor competing for adsorption sites of NO_2_ on the surface of Cr_2_WO_6_ nanoparticles. When the humidity reached 4% RH at 25°C, sensor resistance was promoted by ~2 times and the sensor responses were enhanced in the humid conditions in Figure 4. The sensor response was increased by almost 2 times to 2 ppm NO_2_ in the presence of 4% RH at 25°C. In the sensing processes, NO_2_ acted as the acceptor, meanwhile the water vapor was the donor. Moreover, the water vapor gave an inhibition to the oxygen adsorption on the material surface. Interestingly, the sensor responses were reduced when the humidity increased to 14 and 20% RH at 25°C. That may owe to the chemical adsorption of water vapor, which occupies the adsorption sites of NO_2_. However, the responses were still higher than that in dry conditions. Moreover, the sensing properties balanced with N_2_ in humid atmosphere were also analyzed by Keithley multimeter; nevertheless, the water vapor gave a promotion to sensor resistance compared with that in dry conditions. Therefore, we cannot evaluate the performance of the sensor devices based on the existing equipment in the laboratory.

**Figure 5:**
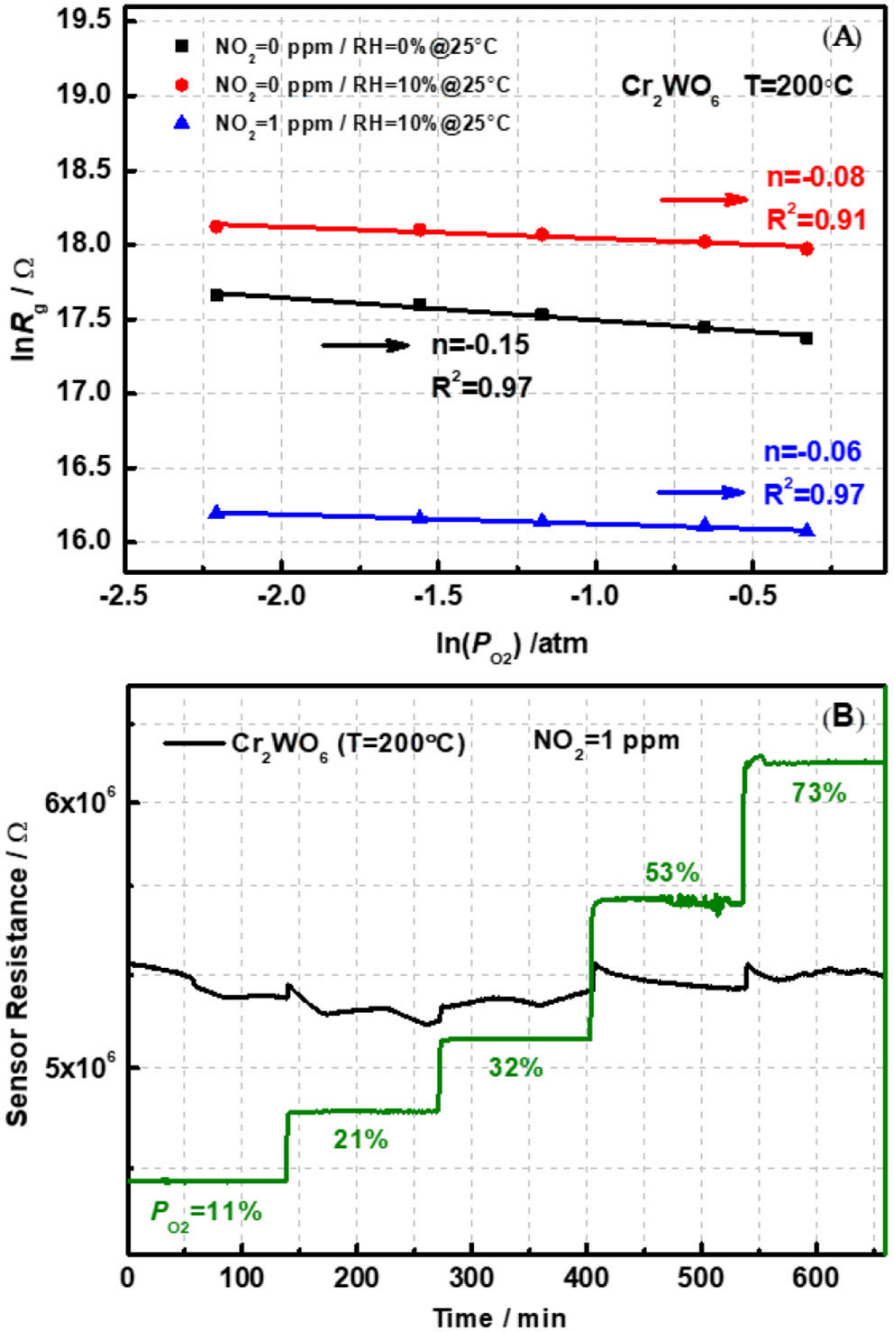
**(A)** The power-law response to oxygen for pristine Cr_2_WO_6_ in the absence and presence of water vapor and **(B)** a transient response to O_2_ in the presence of 1 ppm NO_2_ at 200°C.

According to our previous reports, oxygen adsorption, and reaction play a basic role in the sensing process with the formation of ${\text{O}}_{2}^{-}$, O^−^, or O^2−^ on the surface of metal oxides (Hua et al., 2018a,b,c). The oxygen adsorption behavior could be analyzed by the relationship between sensor resistance (*R*_g_) and partial pressure of oxygen (*P*_O2_) in different atmospheres, i.e., power-law response. Figure 5 shows the linear plot of *R*_g_ on the *P*_O2_ ranging from 0.1 to 0.7 atm (1 atm = 100% in volume) in a double logarithm-scale at an operation temperature of 200°C. It was obvious that the *R*_g_ decreased with increasing *P*_O2_, indicating the presence of oxygen adsorption on the surface of Cr_2_WO_6_ nanoparticles. Adsorption of oxygen on the surface traps electrons and releases holes resulting in a reduction in sensor resistance. Moreover, Figure 5A presents the power-law response to oxygen, and the fitting slope (*n*) was −0.15 at 200°C. However, for p-type materials, the receptor and transducer functions have not been built yet. Thus, the fitting power-law exponent could not be well-clarified. However, it was quite clear that oxygen adsorption served as the receptor function for Cr_2_WO_6_ without difference with typical n-type WO_3_ or p-type Cr_2_O_3_ (Hua et al., 2018a,b,c). As mentioned before, water vapor forms chemical adsorption on surface leading to a block on oxygen adsorption as shown in Figure 5A. With humidity increasing from 0 to 10% RH at 25°C, *R*_g_ was promoted at all *P*_O2_ and the absolute value of *n* was decreased, suggesting a strong electronic interaction of oxygen with the Cr_2_WO_6_ surface. This may be caused by the competitive adsorption between oxygen and water molecules (Hua et al., 2018c). With the presence of NO_2_, the power-law response to oxygen was also investigated to clarify the role of oxygen on the sensing process of NO_2_ (Hua et al., 2018d). When exposed to NO_2_ of 1 ppm, the sensor resistance greatly decreased. According to Figure 5B, the power-law response to oxygen is not linear, suggesting that the adsorption of oxygen is influenced by NO_2_ adsorption. More specifically, the electronic interaction of oxygen with the Cr_2_WO_6_ surface was significantly inhibited by NO_2_, i.e., shielded by NO_2_. This could be due to a competitive adsorption of NO_2_, which may share the same adsorption sites, in case of typical MOS, are metal atoms and oxygen vacancies. When exposed to humidity in the presence of NO_2_ (1 ppm balanced with N_2_), sensor resistance was almost independent of *P*_O2_, and the power-law exponent was merely 0.06, which is much smaller than that in a dry condition. The corresponding transient responses of power law response were shown in Figure S6 of Supplementary Material D. In addition, it was noted that the absolute value of the power-law exponent, *n* became much smaller compared with that in the absence of NO_2_. In other words, humidity not only promotes the sensing response but also changes the basic mechanism of p-type Cr_2_WO_6_ to NO_2_ according to the power-law response. It is possible that in the presence of humidity oxygen adsorption may take place with a competitive adsorption with water, which forms a weak adsorption on the surface of Cr_2_WO_6_. This causes a weak adsorption.

## Conclusion

A new sensing material Cr_2_WO_6_ was prepared and its sensing properties to NO_2_ were investigated in both dry and humid conditions. The basic sensing mechanism was also analyzed by surface oxygen adsorption behavior in different atmospheres. Here are the conclusions:

➢ Sensors based on Cr_2_WO_6_ nanoparticles showed great response to NO_2_ based on air atmosphere down to 0.2 ppm. The sensor responses in N_2_ conditions were higher than in air conditions over 1 ppm of NO_2_.
➢ Sensor resistances were raised up with the relative humidity increasing. However, sensor responses to all gases were enhanced by water vapor over the range of 0–20% RH at 25°C.
➢ The oxygen adsorption played a vital basic role in the sensing process. However, the oxygen adsorption behavior was inhibited by the presence of water vapor and NO_2_.

In conclusion, sensing material Cr_2_WO_6_ shows a great response to detect NO_2_ in dry and humid conditions.

## Data Availability Statement

All datasets generated for this study are included in the article/Supplementary Material.

## Author Contributions

MY and CT performed the experiments and analyzed the data with the help from YW, YL, and ZH. YW and ZH conceived and modified the manuscript based on experimental data.

### Conflict of Interest

YL was employed by the Beijing Institute of Mechanical Equipment that is a government-owned non-profit organization. The remaining authors declare that the research was conducted in the absence of any commercial or financial relationships that could be construed as a potential conflict of interest.

## References

[B1] Afzal A. Cioffi N. Sabbatini L. Torsi L (2012). NO_x_ sensors based on semiconducting metal oxide nanostructures: Progress and perspectives. Sens. Actuators B Chem. 171, 25–42. 10.1016/j.snb.2012.05.026

[B2] Bârsan N. Simion C. Heine T. Pokhrel S. Weimar U. (2010). Modeling of sensing and transduction for p-type semiconducting metal oxide based gas sensors. J. Electroceram. 25, 11–19. 10.1007/s10832-009-9583-x

[B3] Bodneva V. Ilegbusi O. Kozhushner M. Kurmangaleev K. Posvyanskii V. Trakhtenberg L. (2019). Modeling of sensor properties for reducing gases and charge distribution in nanostructured oxides: a comparison of theory with experimental data. Sens. Actuators B Chem. 287, 218–224. 10.1016/j.snb.2019.02.034

[B4] Choi Y. Sakai G. Shimanoe K. Yamazoe N. (2004). Wet process-based fabrication of WO_3_ thin film for NO_2_ detection. Sens. Actuators B Chem. 101, 107–111. 10.1016/j.snb.2004.02.031

[B5] Hoa N. Quy N. Kim D. (2009). Nanowire structured SnO_x_-SWNT composites: high performance sensor for NO_x_ detection. Sens. Actuators B Chem. 142, 253–259. 10.1016/j.snb.2009.07.053

[B6] Hua Z. Li Y. Zeng Y. Wu Y. (2018a). A theoretical investigation of the power-law response of metal oxide semiconductor gas sensors I: Schottky barrier control. Sens. Actuators B Chem. 255, 1911–1919. 10.1016/j.snb.2017.08.206

[B7] Hua Z. Qiu Z. Li Y. Zeng Y. Wu Y. Tian X. (2018b). A theoretical investigation of the power-law response of metal oxide semiconductor gas sensors II: Size and shape effects. Sens. Actuators B Chem. 255, 3541–3549. 10.1016/j.snb.2017.09.189

[B8] Hua Z. Tian C. Huang D. Yuan W. Zhang C. Tian X. (2018c). Power-law response of metal oxide semiconductor gas sensors to oxygen in presence of reducing gases. Sens. Actuators B Chem. 267, 510–518. 10.1016/j.snb.2018.04.002

[B9] Hua Z. Tian C. Qiu Z. Li Y. Tian X. Wang M. (2018d). An investigation on NO_2_ sensing mechanism and shielding behavior of WO_3_ nanosheets. Sens. Actuators B Chem. 259, 250–257. 10.1016/j.snb.2017.12.016

[B10] Jaegle L. Steinberger L. Martin R Chance K. (2005). Global partitioning of NO_x_ sources using satellite observations: relative roles of fossil fuel combustion, biomass burning and soil emissions. Faraday Discuss. 130, 407–423. 10.1039/b502128f 16161795

[B11] Kida T. Nishiyama A. Yuasa M. Shimanoe K. Yamazoe N. (2009). Highly sensitive NO_2_ sensors using lamellar-structured WO_3_ particles prepared by an acidification method. Sens. Actuators B Chem. 135, 568–574. 10.1016/j.snb.2008.09.056

[B12] Ling Z. Leach C. (2004). The effect of relative humidity on the NO_2_ sensitivity of a SnO_2_/WO_3_ heterojunction gas sensor. Sens. Actuators B Chem. 102, 102–106. 10.1016/j.snb.2004.02.017

[B13] Marichy C. Donato N. Latino M. Georg Willinger M. Tessonnier J.-P. Neri G.. (2015). Gas sensing properties and p-type response of ALD TiO_2_ coated carbon nanotubes. Nanotechnology 26:024004. 10.1088/0957-4484/26/2/024004 25525827

[B14] Nguyen D. El-Safty S. (2011). Synthesis of mesoporous NiO nanosheets for the detection of toxic NO_2_ gas. Chem. Eur. J. 17, 12896–12901. 10.1002/chem.201101122 21739494

[B15] Richter A. Burrows J. Nüß H. Granier C. Niemeier U. (2005). Increase in tropospheric nitrogen dioxide over China observed from space. Nature 437, 129–132. 10.1038/nature04092 16136141

[B16] Stǎnoiu A. Simion C. Diamandescu L. Tǎrǎbǎşanu-Mihǎilǎ D. Feder M. (2012). NO_2_ sensing properties of Cr_2_O_3_ highlighted by work function investigations. Thin Solid Films 522, 395–400. 10.1016/j.tsf.2012.09.003

[B17] Sun W. Shao M. Granier C. Liu Y. Ye C. Zheng J. (2018). Long-term trends of anthropogenic SO_2_, NO_x_, CO, and NMVOCs emissions in China. Earths Future 6, 1112–1133. 10.1029/2018EF000822

[B18] Vyas R. Sharma S. Gupta P. Prasad A. K. Dhara S. Tyagi A. K. (2013). Nitrogen dioxide induced conductivity switching in ZnO thin film. J. Alloys Comp. 571, 6–11. 10.1016/j.jallcom.2013.03.217

[B19] Zhou W. Huang J. Li J. Xu Z. Cao L. Yao C. (2015). Cr_2_WO_6_ nanoparticles prepared by hydrothermal assisted method with selective adsorption properties for methylene blue in water. Mater. Sci. Semicond. Process. 34, 170–174. 10.1016/j.mssp.2015.02.010

