# Supplementary material for: NO2 Sensing Properties of Cr2WO6 Gas Sensor in Air and N2 Atmospheres
Source: Front Chem. 2020 Jan 23;7:907. doi: 10.3389/fchem.2019.00907 (PMC6989548; doi:10.3389/fchem.2019.00907)
Supplement: Supplementary file 1 [file Data_Sheet_1.pdf]

Supplementary Material

Supplementary material A: Materials and Sensing Measurements

Chromium(III) nitrate nonahydrate (99.95%) and sodium tungstate dehydrate (ACS 99.0%–101.0%) were provided by Shanghai Aladdin Biochemical Technology Co., Ltd. Chromium tungstate (Cr_2WO_6) nanoparticles were synthesized by a hydrothermal assisted process. Typically, the amount of raw material was calculated according to a molar ratio 2:1 of chromium salt ions to tungstate, and then was dispersed in deionized water with the magnetic stirring, respectively. Then, the solution was mixed and neutralized to pH=5 by adding sodium hydroxide and kept stirring for 4 h. The resulting suspension was performed with hydrothermal treatment at 200ºC for 24 h. Finally, it was given a centrifugation for 3 times and dried at 80ºC for 12 h, and annealed at 1000ºC in air for 2 h with a heating rate of 5ºC/min.

The sample powders was mixed with glycerin to form a homogeneous paste. The paste was screen-printed on the alumina substrate that consisted of a pair of comb-type Au microelectrodes and micro Pt heater on the backside as shown in Figure S1. Then devices were dried at 80ºC and calcined at 500ºC in air for 2 h. Finally, the alumina substrate was wire-bonded onto a hexapod socket special for gas sensor (Suzhou IST Co., Ltd.). Figure S1 showed the structure diagram and cross-section view of sensor devices. In order to obtain good stability, all sensors were aged at 400ºC for 24 h before performing tests.

The gas sensors performance was measured by DC resistance with a homemade apparatus equipped with a dynamic gas distribution system as shown schematically in Figure 1 and Figure S2. The sensor devices were placed in the polytetrafluoroethylene cavity provided with a gas inlet and a gas outlet. The operating temperature was controlled by an adjustable DC power supply (DP832, RIGIO, China) and calibrated by an infrared camera (Optris, PI200, Germany). The gas flow rate was dominated with a constant rate of 100 cm^3/min using mass flow controllers (MFCs). Target gases were supplied by gas cylinders with appropriate concentrations balanced with the carrier gases (air or nitrogen). The sensors properties were performed at 200-350ºC in both dry and humid conditions. The humidity and oxygen concentrations were calibrated using a humidity sensor (SHT31-ARP, Sensirion, Switzerland) and oxygen analyzer (SST, England), respectively. A LabVIEW (National Instruments) interface based on a Keithley multimeter (Keithley 2000, USA) was used to collect all data to computer in real time continuously.

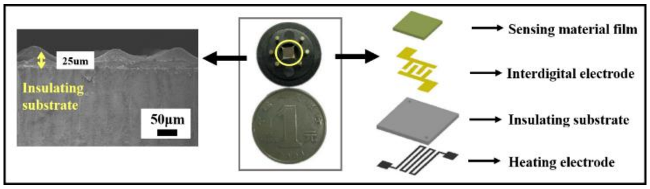

Figure S1. Schematic drawing and photograph of the sensor devices using in the present work.

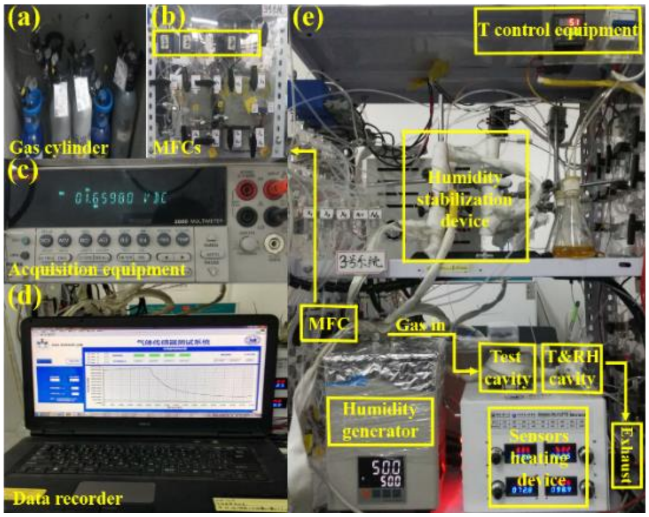

Figure S2. The whole and all parts of experimental set-up for the characterizations of gas sensing properties.

Supplementary material B: XRD Patterns for Nanoparticles

Figure S3 was the XRD spectra of powders that is fitted with the tetragonal phase of Cr_2WO_6 (JCPDS 35-0791). The obtained powders annealed at 1000ºC were giving narrow peaks and performed no other peaks that suggested great crystal quality and high purity.

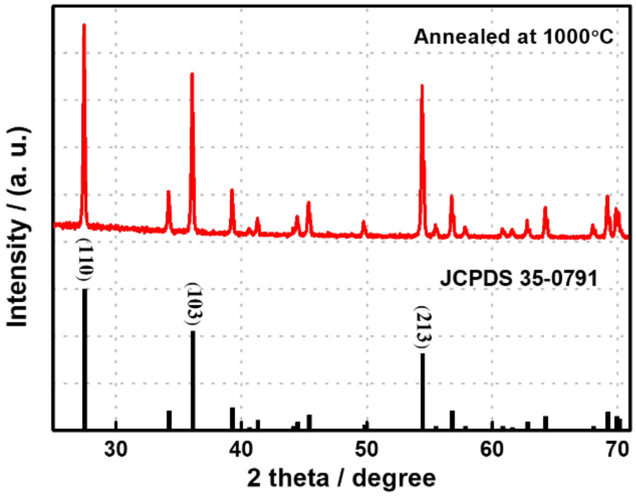

Figure S3. XRD patterns of pristine $CrWO_6$ nanoparticles powders.

Supplementary material C: The Transient Response to NO_2 in N_2 Atmosphere and the Sensing Properties in Humid Conditions

Figure S4 was the transient responses to NO_2 balanced with N_2 at 200ºC-350ºC from 0.2 to 5 ppm. The transient responses to NO_2 balanced with air in the presence of water vapor were presented in Figure S5 with the range of 0-20% RH @25ºC.

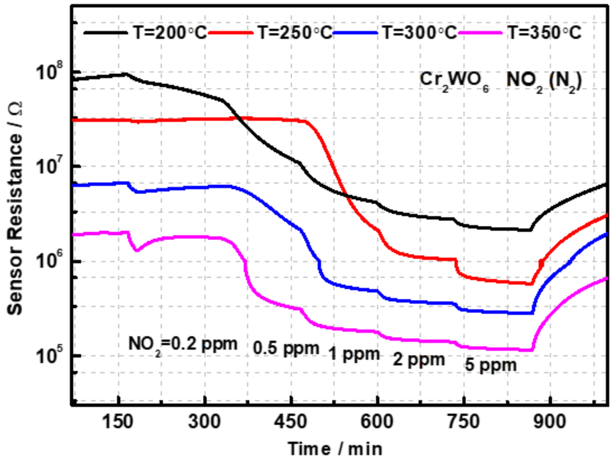

Figure S4. The transient sensor responses to NO_2 balanced with N_2 in dry conditions from 250ºC to 350ºC.

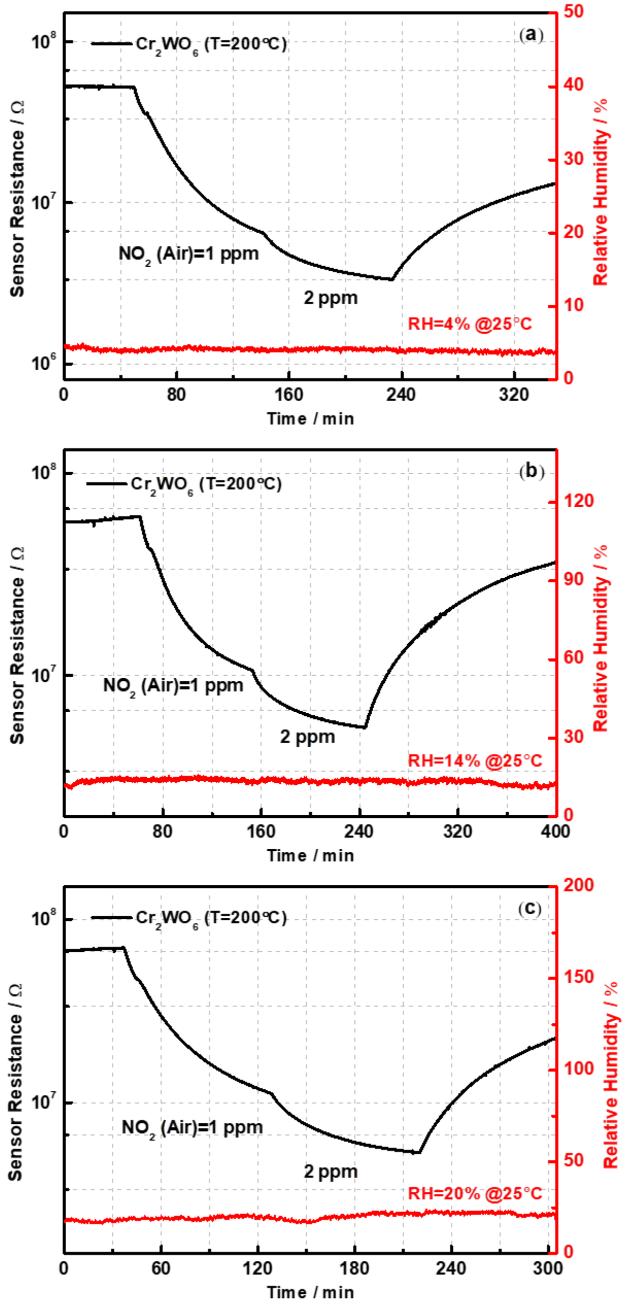

Figure S5. The transient response of 1 and 2 ppm of NO_2 balanced with air in humid atmosphere with the range of 0-20% RH @25ºCat 200ºC.

Supplementary material D: Transentitent Response of Power-law Response

Figure S6 presented the sensor resistance with differernt oxygen conentration at 200ºC. In addition, the resistance in humid conditions was also investigated as shown in Figure S6b and Figure S6c. The sensor resistance was measured in the presence of both oxidizing gas and water vapor.

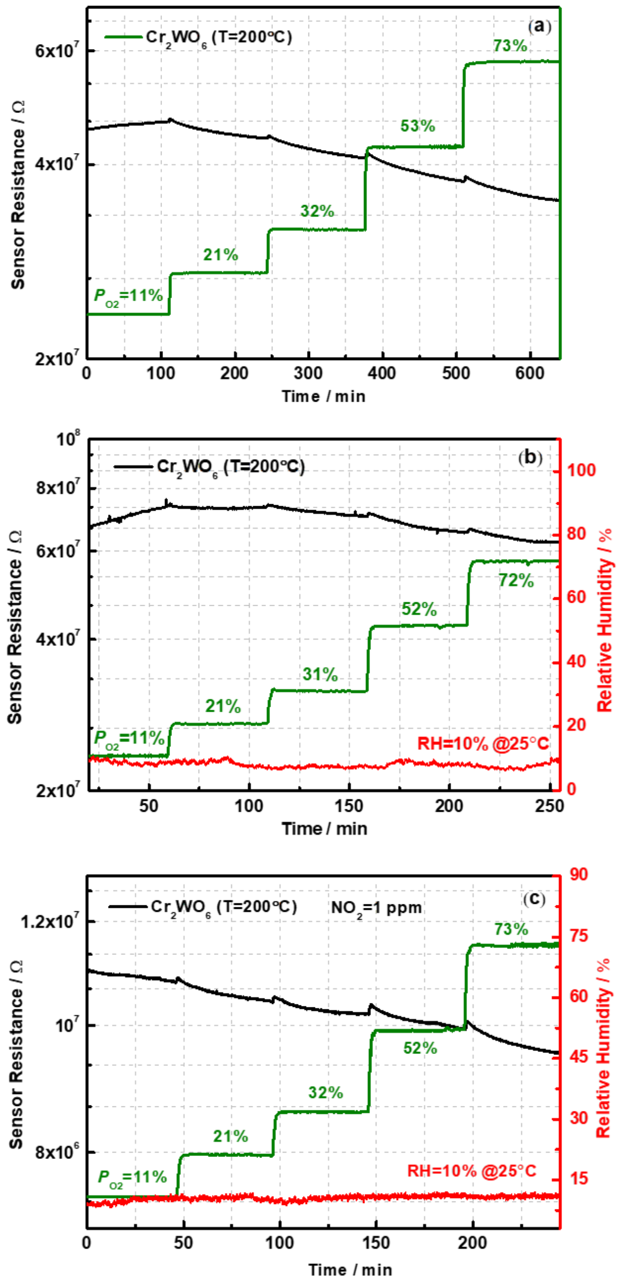

Figure S6. The transient responses to oxygen of Cr_2WO_6 in (a) dry conditions, (b) and (c) in the absence and presence of 1 ppm NO_2 of humid conditions.

Supplementary material E: The Stability of Sensors

Figure S7 presented the sensors transient resistance in the presence of 5 ppm NO_2 balanced with air at 300ºC, which last 20 hours and 4 times of repeated tests.

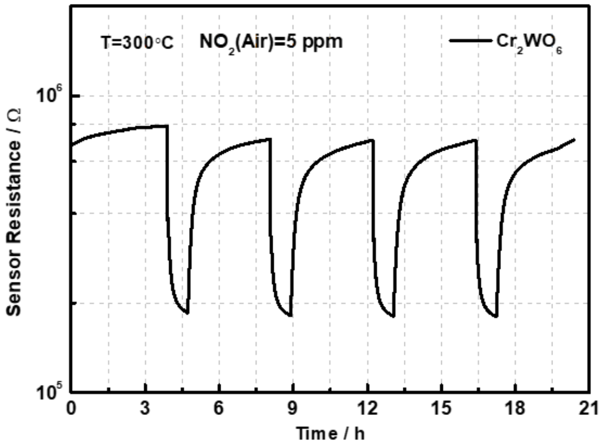

Figure S7. The repeating transient responses to NO_2 balanced with air of 5 ppm at 300ºC.
